# Radiomic signatures from postprocedural MRI thalamotomy lesion can predict long-term clinical outcome in patients with tremor after MRgFUS: a pilot study

**DOI:** 10.3389/fradi.2025.1683274

**Published:** 2025-11-06

**Authors:** Antonio Innocenzi, Sara Peluso, Federico Bruno, Laura Balducci, Ettore Rocchi, Michela Bellini, Alessia Catalucci, Patrizia Sucapane, Gennaro Saporito, Tommasina Russo, Gastone Castellani, Francesca Pistoia, Alessandra Splendiani

**Affiliations:** 1 Department of Biotechnological and Applied Clinical Sciences, University of L’Aquila, L'Aquila, Italy; 2 Department of Medical and Surgical Sciences, University of Bologna, Bologna, Italy; 3 IRCCS Azienda Ospedaliero Universitaria di Bologna, Bologna, Italy; 4 Neuroradiology, San Salvatore Hospital, L’Aquila, Italy; 5 Department of Life, Health and Environmental Sciences, University of L’Aquila, L'Aquila, Italy; 6 Neurology, San Salvatore Hospital, L’Aquila, Italy

**Keywords:** essential tremor, Parkinson's disease, MRgFUS thalamotomy, machine learning, radiomics, MRI, neuroimaging

## Abstract

**Objective:**

Magnetic resonance-guided focused ultrasound (MRgFUS) thalamotomy is an effective treatment for essential tremor (ET) and tremor-dominant Parkinson's disease (PD), yet a substantial proportion of patients experience tremor recurrence over time. Reliable imaging biomarkers to predict long-term outcomes are lacking. The purpose of the study was to evaluate whether radiomic features extracted from 24-h post-treatment MRI can predict clinically relevant tremor recurrence at 12 months after MRgFUS thalamotomy, using a machine learning (ML) approach.

**Materials and methods:**

Retrospective, single-center study included 120 patients (61 ET, 59 PD) treated with unilateral MRgFUS Vim thalamotomy between February 2018 and June 2023. Tremor severity was assessed using part A of the Fahn–Tolosa–Marin Tremor Rating Scale (FTM-TRS) at baseline and 12 months. Recurrence was defined as an FTM-TRS part A score ≥ 3 at 12 months. Lesions were manually segmented on 24-h post-treatment T2-weighted MRI. Forty radiomic features (18 first-order, 22 texture GLCM from Laplacian of Gaussian–filtered images) were extracted. A linear Support Vector Classifier with leave-one-out cross-validation was used for classification. Model explainability was assessed using SHapley Additive exPlanations (SHAP).

**Results:**

Clinically relevant tremor recurrence occurred in 23 patients (19%). For the full cohort, the ML model achieved a balanced accuracy of 0.720, weighted F1-score of 0.737, and comparable sensitivity and specificity across classes. Performance was higher in PD (BA = 0.808, F1 = 0.793) than in ET (BA = 0.580, F1 = 0.696). The most predictive features were texture-derived GLCM metrics, particularly from edge-enhanced images, with first-order features contributing complementary information. No significant correlations were found between radiomic features and procedural parameters.

**Conclusion:**

Radiomic analysis of MRgFUS lesions on 24-h post-treatment MRI can provide early prediction of 12-month tremor recurrence, with higher predictive value in PD than in ET. Texture-based features may capture microstructural characteristics linked to treatment durability. This approach could inform post-treatment monitoring and individualized management strategies.

## 1 Introduction

Magnetic resonance–guided focused ultrasound (MRgFUS) thalamotomy targeting the ventral intermediate nucleus (Vim) of the thalamus has emerged as an innovative, minimally invasive treatment for essential tremor (ET) and tremor-dominant Parkinson's disease (PD) (1). Long-term follow-up studies, with data extending beyond five years, consistently demonstrate immediate and substantial tremor reduction, as measured by clinical rating scales and disability scores (2, 3).

Despite these encouraging results, tremor recurrence remains a concern. A recent meta-analysis in ET patients reported that, at 4–5 years after treatment, tremor recurrence occurred in approximately 23%–25% of cases (4). Similarly, a retrospective study in tremor-dominant PD reported a 23% relapse rate even within the first month (5). Most recurrences were partial, with tremor severity still improved compared with baseline in most patients. Nonetheless, these findings highlight the importance of identifying reliable predictors of sustained, long-term benefit.

Several studies have investigated potential prognostic factors for treatment failure or recurrence. Along with confirming higher relapse rates in PD, previous research has examined procedural parameters (e.g., number of sonications, maximum temperature achieved), lesion morphology on post-treatment MRI (e.g., size, shape), and advanced imaging markers from diffusion tensor imaging (DTI) and tractography of the dentato–rubro–thalamic pathway at the Vim level (5–9). However, results have been inconsistent, and no reliable method currently exists to predict the long-term durability of MRgFUS outcomes.

Radiomics and machine learning (ML) applied to MRI enable extraction of high-dimensional quantitative features—many imperceptible to the human eye—that may serve as imaging biomarkers. In oncology, multiple studies have demonstrated the prognostic value of imaging-derived radiomic features in treatment-induced thermal ablation lesions (10–12).

Radiomics and ML have been also applied to classify clinical characteristics of ET and PD, differentiate ET from healthy controls, explore radiomic correlations of clinical variables in ET, and distinguish PD motor subtypes (13–19). The results of previous studies provide preliminary evidence that radiomics analysis represents a growing potential imaging biomarker for both diagnosis and prognosis in movement disorders.

To the best of our knowledge, no prior study has applied radiomic analysis to MRgFUS thalamotomy lesions. This study addresses that gap by using MRI-derived radiomic biomarkers and ML to predict tremor relapse after treatment. We conducted a retrospective, single-center study to develop an ML pipeline for predicting 12-month tremor recurrence using radiomic features extracted from lesion segmentations on MRI performed 24 h after MRgFUS. We also quantitatively evaluated the contribution of individual radiomic features to model performance.

## 2 Materials and methods

### 2.1 Participants

We retrospectively evaluated 120 patients who underwent unilateral MRgFUS Vim thalamotomy at a single center between February 2018 and June 2023. Details of the procedure have been described elsewhere (1).

From clinical records, we extracted demographic data (sex, age) and clinical information, including underlying diagnosis (ET or PD) and tremor intensity using part A of the Fahn–Tolosa–Marin Tremor Rating Scale (FTM-TRS). According to our clinical protocol, all patients underwent evaluation before treatment, the day after the procedure, and at follow-up visits scheduled at 1 month, 6 months, 1 year, and 2 years.

Tremor severity at 12 months was assessed with the FTM-TRS. Scores were binarized to indicate the presence or absence of clinically relevant tremor recurrence, framing the task as binary classification. Patients with a total FTM-TRS part A score < 3 were assigned to class 0 (no clinically significant tremor), while those with a score ≥ 3—considered a clinically relevant recurrence—were assigned to class 1. This threshold was selected based on clinical reasoning and it represents the lowest value which could interfere with the Quality of Life (QoL) of the patients as described by the same authors of the method *Clinical rating scale for tremor* (20). Moreover, as stated by Braccia et al. in (5), 30% of tremor intensity recurrence could be a reasonable cut off. The binarization yielded 97 patients in class 0 and 23 in class 1. Regarding the exact relapse rates for the two different diagnoses, within class 0, 44 patients were in the PD group and 53 patients were in the ET group, while within class 1 there were 15 PD patients and 8 ET patients.

From procedural reports, we recorded the total number of sonications, the number reaching ≥ 50°C, and the number reaching ≥ 54°C.

Exclusion criteria were: (i) incomplete clinical records; (ii) tremor not attributable to PD or ET; and (iii) absence of FTM-TRS part A (upper limbs) data at 12 months post-treatment.

### 2.2 Image acquisition and segmentation

In our Institutional protocol, all patients undergo brain MRI the day after the procedure. All MRI scans were acquired on a 3 T scanner (Discovery 750; GE Healthcare, Milwaukee, WI, USA) with a 32-channel head coil. The imaging protocol included:

- Axial FLAIR: slice thickness = 3.0 mm, interslice gap = 0.3 mm, TR = 11,000 ms, TE = 125 ms, inversion time (TI) = 2,800 ms, frequency FOV = 240 mm, phase FOV = 0.8.
- Axial GRE: slice thickness = 3.0 mm, interslice gap = 0.3 mm, TR = 960 ms, TE = 25 ms, flip angle = 20°, frequency FOV = 260 mm, phase FOV = 0.75.
- Axial SWI: slice thickness = 2.0 mm, TR = 49 ms, TE = 40 ms, frequency FOV = 240 mm, phase FOV = 0.85.
- Axial DWI: slice thickness = 3.0 mm, interslice gap = 0.3 mm, TR = 10,550 ms, TE = 85 ms, b-values = 0 and 1,000 s/mm^2^, frequency FOV = 260 mm, phase FOV = 0.8.
- Axial and Coronal T2 FSE: slice thickness = 3.0 mm, interslice gap = 0.3 mm, TR = 7,854 ms, TE = 85 ms, frequency FOV = 260 mm, phase FOV = 0.8; acquired in axial and coronal planes.
- 3D T1-weighted IR-FSPGR: isotropic voxel size = 1.0 mm^3^, slice thickness = 1.0 mm, TR = 8.5 ms, TE = 3.2 ms, inversion time = 450 ms, flip angle = 12°, frequency FOV = 256 mm, phase FOV = 0.8; reconstructed in axial, coronal, and sagittal planes.

For the purpose of the study, T2W FSE images were segmented. Anonymized Digital Imaging and Communications in Medicine (DICOM) files were retrieved from the hospital PACS and converted to Neuroimaging Informatics Technology Initiative (NIfTI) format for radiomic analysis. The Region of Interest (ROI) was defined as the MRgFUS-induced Vim lesion, including surrounding edema.

**Figure 1:**
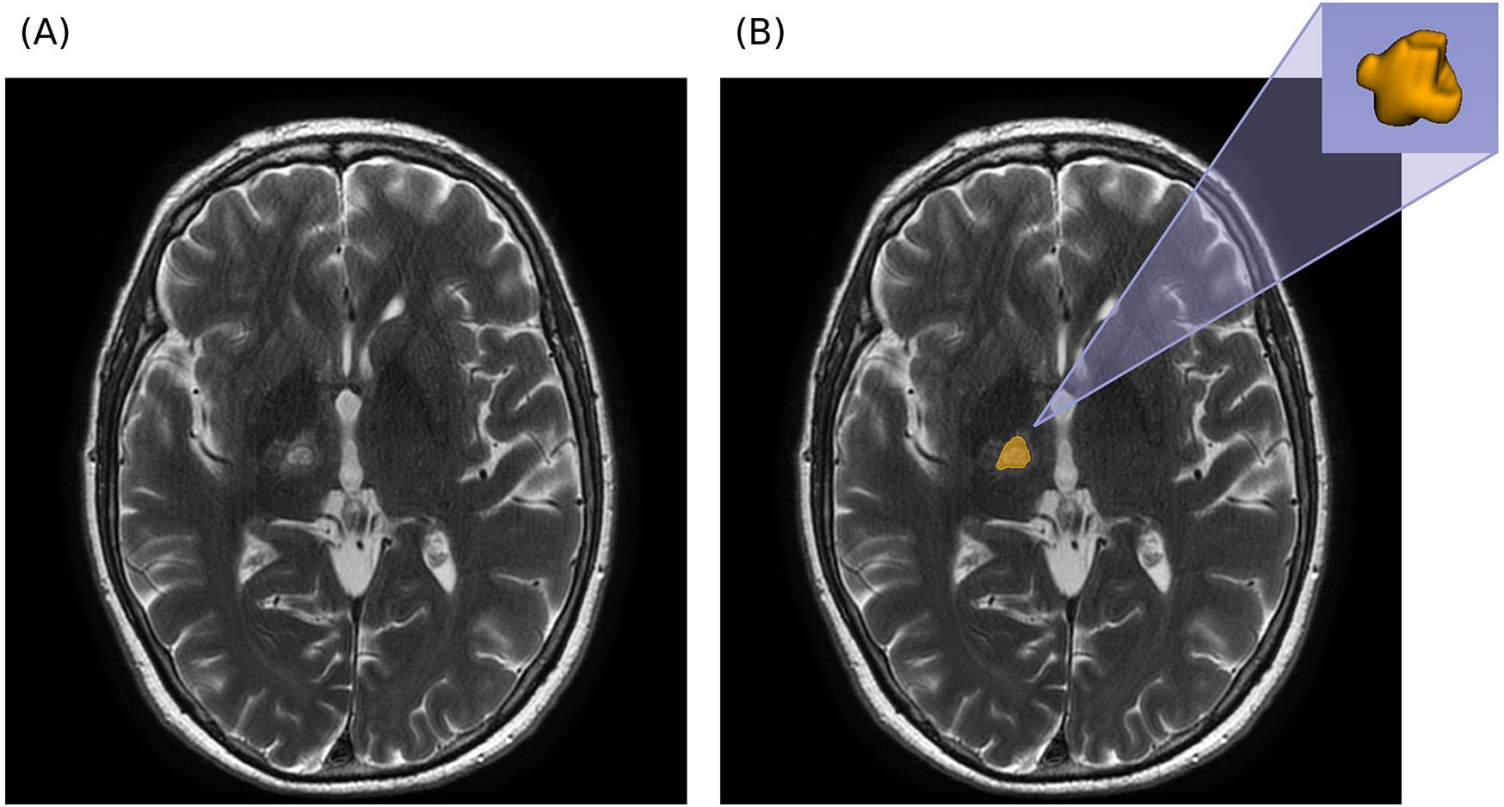
Segmentation example in axial T2W MRI. **(A)** Axial MRI prior to segmentation. **(B)** Axial MRI with overlapping segmentation mask and 3D reconstruction of segmented ROI (obtained with 3D Slicer).

Manual segmentation was performed by experienced radiologists using 3D Slicer (v5.6.2) (21, 22). A median smoothing filter with a spherical kernel radius of 3.0 mm was applied to the segmentation mask before export to reduce minor surface irregularities and mitigate inter-operator variability. An example of the segmentation and 3D ROI reconstruction is shown in Figure 1.

### 2.3 Feature extraction

Radiomic features were extracted using the PyRadiomics package (v3.1.0) in Python (v3.9) operating in 3D (23). Workflow automation was managed with Snakemake (v7.32.4) (24).

Feature extraction was customized by specifying settings for pre-processing operations on MR images, by specifying the feature classes to extract, and by enabling the use of specified filters on the MR image. Regarding settings for pre-processing, MR images underwent image normalization (meaning that Z-score standardization was applied to voxels, based on all grey values in the image), and images were resampled to an isotropic spacing of 1 mm3 using a B-spline interpolator. Geometry tolerance for comparison of origin, direction and spacing between the image and the mask was set to 1 × 10-2. Finally, bin width for discretization of the image gray level intensities was set to 10. In terms of feature classes and filters applied on the image, first order features were extracted on the original (unfiltered) image, while second order features, namely Grey Level Co-occurrence Matrix (GLCM, Haralick) features, were extracted on the image filtered with a Laplacian of Gaussian (LoG) filter to enhance edges and fine details in the ROI (25). Specifically, first order statistics describe the distribution of grey level intensities of voxels in the ROI, while GLCM features describe ROI texture by measuring the occurrences of pairs of voxel intensities in a specific spatial relationship. First order features were extracted on unfiltered images to capture the underlying intensity distribution of tissue in the ROI without alteration of grey level values by filters. A *σ* = 1.5 mm for the LoG filter was selected as a balanced compromise between enhancement of fine textural details and preservation of larger-scale structures. This configuration of the filter captures both local texture variations and broader structural patterns. Feature extraction included first-order statistics from the original (unfiltered) images, capturing the distribution of voxel intensities, and Gray Level Co-occurrence Matrix (GLCM) features from images filtered with a Laplacian of Gaussian (LoG) filter (*σ* = 1.5 mm), enhancing both fine textural details and larger-scale structures. This process yielded 40 features: 18 first-order and 22 GLCM.

### 2.4 Classification pipeline

Radiomic features were used as input to a binary classification pipeline having as target the 12-month FTM binarized value. Given the moderate sample size (120 patients) relative to the number of radiomic features (40), a linear Support Vector Classifier (SVC) was selected for its ability to handle moderately high-dimensional data while maintaining good generalization performance in settings with limited observations. The SVC hyperparameters were selected as follows: a linear kernel, a regularization parameter C = 0.1 for the L2 penalty, and balanced class weight. The model was evaluated via Leave-One-Out Cross-Validation (LOO CV), so that its robustness might be assessed even with a small dataset. The LOO CV trains the model on all samples except one and tests the model on the excluded sample; this process is repeated once per sample, ensuring that each sample serves as the test sample exactly once. Within the pipeline, prior to being fed to the classifier, features were standardized. Other ML classifiers were considered and implemented, including logistic regression, random forest, and gradient boosting classifier. The resulting comparative analysis is reported in the Supplementary Material and provides justification for the choice of linear SVC as final model.

Since the study cohort comprised two pathologies, it was also of interest to assess the model's performance also separately for each pathology using the same trained models. The LOO CV procedure was not repeated on each subset because of the reduced overall sample size, which would result in a further reduced sample size per diagnosis.

Quantitative evaluation of the model was obtained by using Balanced Accuracy (BA), Matthews Correlation Coefficient (MCC), and weighted F1-score as performance metrics. BA evaluates if the model correctly classifies negative cases (class 0) and positive cases (class 1), weighing with respect to the number of samples in each class. BA has range [0, 1]. MCC evaluates how well binary predictions are associated to true labels of data, and it considers all diagnostic errors (true positive TP, true negative TN, false positive FP, false negative FN) and it is particularly suitable to datasets with class imbalance. MCC lies in range [−1, 1], with −1 indicating a completely wrong prediction, 0 a random prediction, and +1 a perfect prediction. F1-score, the harmonic mean of precision and recall, is particularly suitable in biomedical applications as it balances the trade-off between false positives and false negatives, and lies in range [0, 1]. The weighted version of F1-score accounts for class imbalance by weighing each class's F1-score by the number of samples in that class. The confusion matrix was also used for performance assessment; it provides a quick comparison between true labels and predicted labels.

Finally, a key aspect of ML analyses is to provide an interpretation of how models work in producing their predictions—i.e., model explainability. SHAP (SHapley Additive exPlanations) method stands out as one of the most used methods for this purpose: it quantifies the global importance of each input variable while also enabling individual-level interpretation of each variable (26). For these reasons, SHAP was used to provide interpretable explanations for the predictions made by the binary classifier. This approach helps to understand how much each feature contributes to pushing the prediction towards class 0 or class 1, and aggregates feature importance by stacking together all SHAP values from all test samples after the LOO CV.

The classification pipeline was implemented in scikit-learn (v1.5.2) (27), and SHAP values were computed using the shap package (v0.47.0) in Python (v3.12).

## 3 Results

### 3.1 Study population

The cohort included 120 patients (98 males): 61 with ET and 59 with PD. Mean age at treatment was 69 ± 9 years (range: 39–87 years). Disease duration, defined as the interval from tremor onset to MRgFUS treatment, was available for 107 patients (13 missing values) and averaged 14 ± 13 years (range: 1–60 years). Considering the side with the most pronounced tremor, 93 patients had right-sided tremor and 27 had left-sided tremor.

### 3.2 Machine learning performance

The classification task—predicting clinically relevant tremor recurrence at 12 months—was based on binarized FTM scores (Section 2.1), using radiomic features from 24-h post-treatment T2W MRI (Section 2.3) as input to a linear SVC (Section 2.4).

Performance metrics for the Leave-One-Out Cross-Validation (LOO CV) are summarized in Table 1. Results are reported for the entire cohort and for ET and PD subgroups to assess possible pathology-related differences. The highest performance was observed in the PD subgroup, though a predictive signal was also present for ET, reflected by a weighted F1-score of approximately 0.70.

**Table 1:** LOO cross-validated results of ML classification pipeline for binarized FTM RTS part A.

Metrics	Whole cohort	PD	ET
BA	0.720	0.808	0.580
MCC	0.356	0.547	0.113
Weighted F1-score	0.737	0.793	0.696

**Figure 2:**
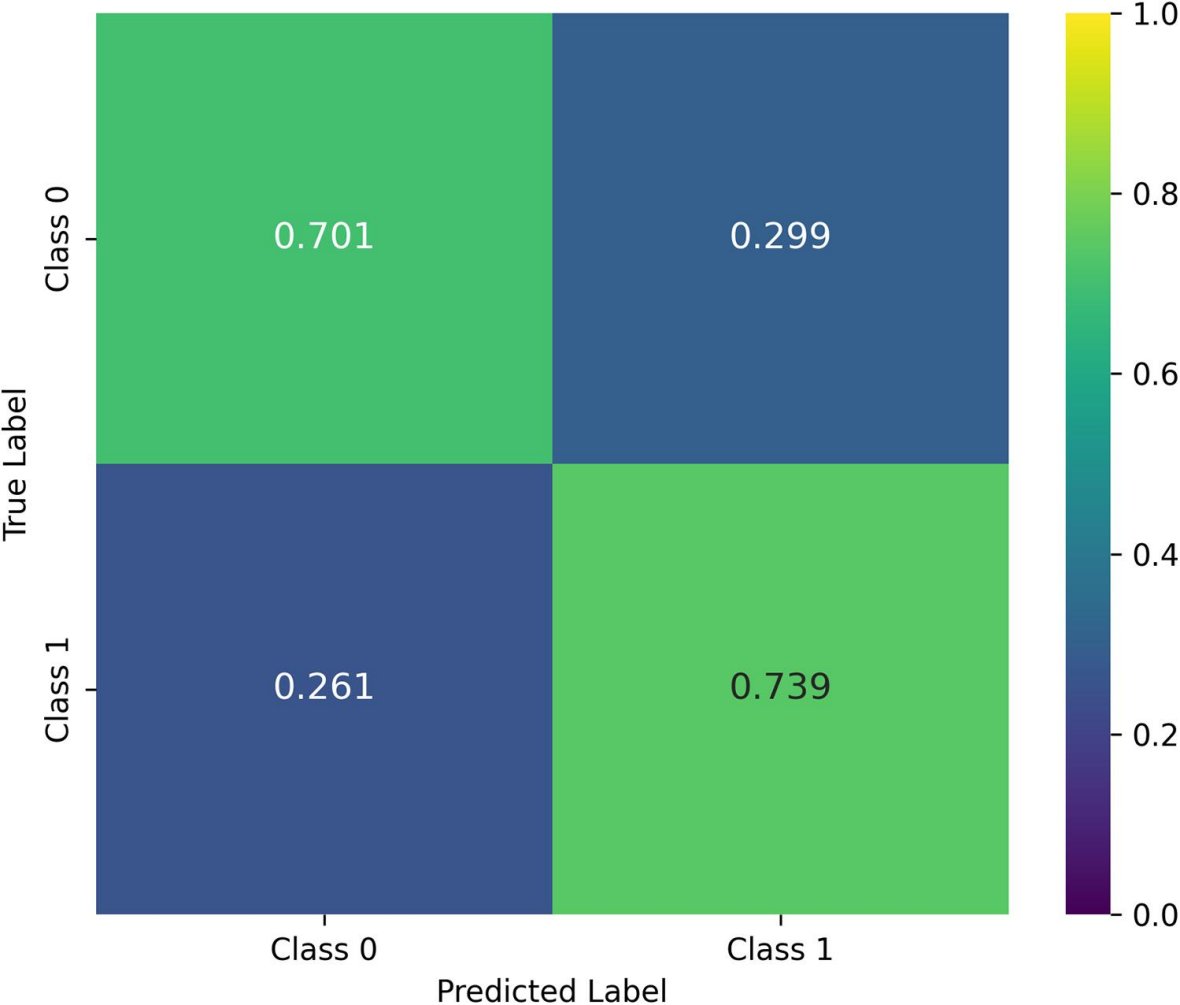
Confusion matrix averaged over cross-validation. The matrix is row-normalized, and reports mean values obtained on each test sample in the LOO CV procedure.

For the entire cohort, the model achieved a balanced accuracy (BA) of 0.720, indicating a good trade-off between sensitivity and specificity. As shown in the confusion matrix (Figure 2), the classifier correctly identified 70.1% of class 0 cases and 73.9% of class 1 cases. The weighted F1-score of 0.737 further demonstrates the model's capacity to manage both classes despite the marked imbalance (81% class 0, 19% class 1). Clinically, this balance is relevant, as both false negatives and false positives carry important implications for tremor management.

### 3.3 Feature importance analysis

**Figure 3:**
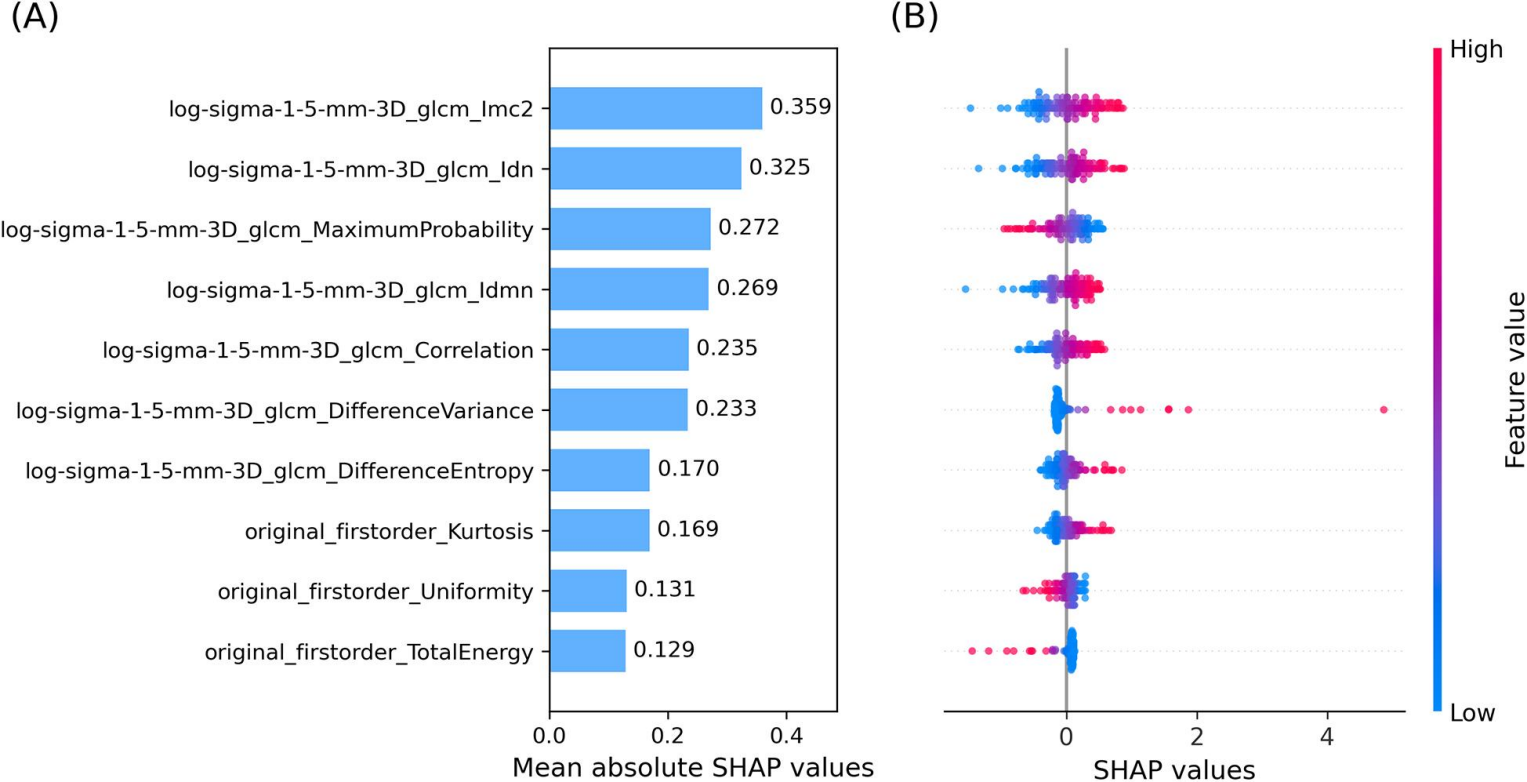
Top ten SHAP values averaged over cross-validation. **(A)** Bar plot of mean absolute SHAP values. **(B)** Beeswarm plot of SHAP values of the top ten input features, averaged across the LOO CV. The colormap links SHAP values and feature values.

Feature contributions were assessed using SHAP values computed within the LOO CV framework. The ten most influential features are shown in Figure 3.

- Panel A: Mean absolute SHAP values averaged over all LOO CV iterations, identifying features with the greatest impact on predictions. The top seven were GLCM-derived from the LoG-filtered image; the remaining three were first-order features from the original image.
- Panel B: Beeswarm plot showing the distribution and direction of SHAP values for each test sample, illustrating how feature variation influenced prediction outcomes.

**Figure 4:**
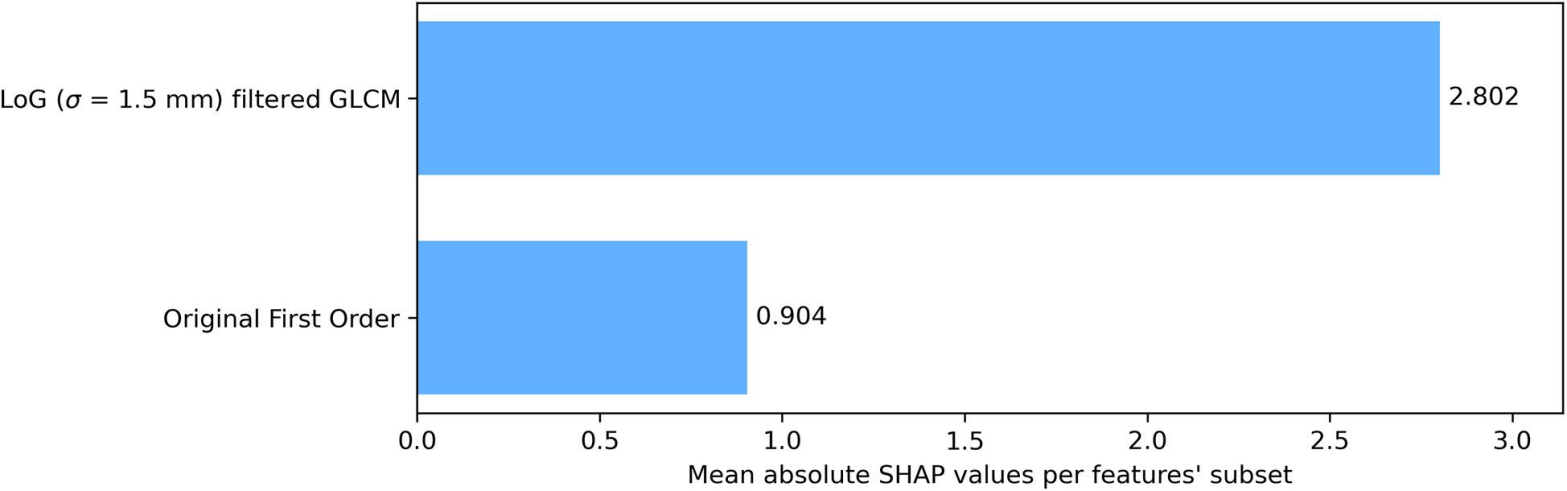
Bar plot of grouped mean absolute SHAP values over cross-validation. Reported values are averaged on each test sample in the LOO CV procedure. Features were grouped according to their category and the image they were extracted on (original or filtered).

To assess category-level contributions, SHAP values were grouped by feature type—GLCM from LoG-filtered images vs. first order from the original image—and averaged across all LOO CV iterations and features within each category (Figure 4). GLCM-LoG features predominated, though first-order features also contributed meaningfully.

### 3.4 Correlation with procedural variables

A Spearman correlation analysis was performed between radiomic features and sonication variables (total number of sonications, number reaching 50°C, number reaching 54°C). After Benjamini–Hochberg correction for multiple comparisons, no statistically significant correlations were found.

### 3.5 Subgroup analysis

When evaluated separately by diagnosis:

- ET: BA = 0.580, MCC = 0.113, weighted F1-score = 0.696
- PD: BA = 0.808, MCC = 0.547, weighted F1-score = 0.793

These results suggest radiomic features from 24-h post-treatment MRI may be more predictive of tremor recurrence in PD than in ET, though a detectable signal exists in both groups.

## 4 Discussion

To the best of the authors' knowledge, this is the first application of a ML model to predict the clinical evolution of a thalamotomy lesion. The application of radiomics in MRgFUS-treated patients with tremor holds promise in predicting 12-month relapse of tremor. This study highlights how a quantitative description of the lesion and edema induced by treatment might be linked to tremor relapse 12 months after treatment, suggesting that short-term tissue response to treatment (24 h after treatment) may already carry be informative for long-term clinical outcomes (12-month tremor relapse). Moreover, as this approach is based on MRI, it offers two key advantages: it enables long-term outcome prediction through a non-invasive imaging modality, and it reduces the impact of operator-dependent variability in MRI interpretation.

Notably, the developed ML pipeline demonstrated a balanced predictive behavior across both FTM classes, as highlighted by comparable classification metrics for each class. This indicates that the model does not favor one class over the other, which is a particularly desirable characteristic in ML models embedded in a clinical context.

Radiomic features analyzed here described both gray-level intensity and texture within the ROI. The combination of first order and second order features was effective for the classification task, reflecting their complementary nature. SHAP analysis indicated that second order GLCM features from edge-enhanced images contributed most to prediction, while first order features provided additional global intensity information.

Referring to the specific features with highest mean absolute SHAP value, the top feature Informational Measure of Correlation 2 (Imc2) quantifies the complexity or uniformity of texture in the ROI of the image by means of correlation between neighboring pixel intensities. It measures the amount of information shared between rows and columns in the normalized GLCM. Higher values of Imc2, meaning less shared information, indicate a more complex and heterogenous texture, while lower values of Imc2 are associated with more shared information and thus a structured, homogeneous texture. Features 2 and 4, Inverse Difference Normalized (Idn) and Inverse Difference Moment Normalized (Idmn) respectively, measure local homogeneity in ROI texture, again highlighting the key role played by the degree of homogeneity of the ROI texture in relapse prediction. Feature 3, Maximum Probability, is the occurrences of the most predominant pair of neighboring grey-level intensity values, quantifying the repetitiveness of a textural pair in the ROI. Features 5–7 measure the degree of local intensity variation and spatial regularity in the ROI: Correlation analyses linear dependency of grey-level values and their respective voxels, Difference Variance focuses on the deviation from the mean of differing grey-level intensity pairs, and Difference Entropy quantifies the randomness in neighborhood grey-level intensity differences. Features 8–10 belong to first order features. Feature 8, Kurtosis, measures the tailedness of the grey-level intensity distribution, thus reflecting the (possible) deviation from a normal intensity distribution. Feature 9, Uniformity, quantifies the homogeneity of grey-level intensities in the intensity distribution (ignoring their spatial distribution, which is instead taken into account by second order features); in other words, it measures how evenly intensities are distributed. Finally, feature 10, Total Energy, reflects the overall signal magnitude within the ROI by calculating the sum of squared intensities and scaling it by the voxel volume in mm^3^.

To our knowledge, this is the first study to use radiomic features from MRgFUS-induced lesions to predict treatment outcome. Prior research has focused primarily on morphological parameters (6). Lesion size and shape remain important predictors: in a prospective cohort of 52 tremor-dominant PD patients, Braccia et al. found that smaller 24-h lesion volumes significantly increased relapse risk, with an optimal range of ∼145–220 mm^3^ for minimizing recurrence. Achieving lesions within this range may balance long-term benefit and adverse-event risk (5). Our previous work also suggested that a more caudally oriented lesion may yield greater stability (28).

Radiomic features may also indirectly reflect histopathological lesion composition. Classically, MRgFUS lesions are described as a necrotic core surrounded by concentric edema zones (6, 29). However, recent histopathological reports suggest preferential demyelination with relative axonal and neuronal preservation.

At the microstructural level, diffusion-based metrics such as fractional anisotropy (FA) within the Vim and along the dentato–rubro–thalamic tract have shown inconsistent correlations with tremor improvement (7, 9, 28). This variability supports integrating multiparametric imaging and raises the possibility that texture-sensitive radiomic features might capture these differences (30).

Our correlation analysis found no association between radiomic features and procedural parameters, consistent with evidence that lesion formation is reliably achieved once temperatures exceed 54°C (7).

It will be of future interest to investigate the applicability of a similar model to the preoperative assessment of the thalamus, with the aim of more accurately selecting patients who are potentially responsive to thalamotomy. A comparable attempt was made by Zhang et al. (31), who applied machine learning techniques to resting-state functional MRI (rs-fMRI) data, identifying that the fractional amplitude of low-frequency fluctuations (fALFF) pattern predicts tremor benefits induced by MRgFUS thalamotomy. Unlike the approach adopted by Zhang et al., an ML-based evaluation of the thalamus in a preoperative setting could explore potential radiomic features capable of predicting responsiveness of the tissues to thalamotomy.

In this direction, Pinheiro et al. (32) reported promising performance of a deep learning model for thalamus segmentation, leaving to future studies the task of achieving a finer segmentation of the thalamus into its distinct nuclei.

The study's strengths include a uniform imaging protocol and systematic post-treatment timing. However, the modest sample size limits generalizability. It will be of future interest to enlarge the study cohort, possibly also by collecting data from different clinical centers to enable a multicentric-scale analysis and external validation of ML models. This would also unlock the possibility of conducting separate analyses for the two pathologies in exam. However, considering the currently available cohort size for the study, techniques to mitigate overfitting of ML models are needed; in particular, LOO CV framework and applying a regularization technique during model training were adopted for this purpose. Moreover, in future studies we would like to evaluate the potential applicability to the entire thalamus in a pre-operative setting to estimate treatment outcome. The available literature suggests that ML can assist clinicians in categorizing patients and achieving more precise diagnoses. Misdiagnosis of ET as other tremor disorders or enhanced physiological tremor remains common in clinical practice. ML has achieved excellent classification performance in the identification of ET, particularly by evaluating features within the cerebello-thalamo-cortical (CTC) pathway (13). Moreover, Panahi et al**.** (15) demonstrated that a radiomic approach may help differentiate Parkinson's disease motor subtypes—tremor-dominant and postural-instability/gait-difficulty—at early stages. Such evaluations are highly relevant in our setting, as more precise clinical diagnoses improve the selection of patients who are more likely to achieve favorable post-procedural outcomes.

Manual segmentation, although protocolized, could benefit from inter-operator variability assessment. Finally, the class imbalance between recurrence and non-recurrence cases may have influenced performance metrics.

Quantitative radiomic analysis of MRgFUS-induced lesions on 24-h post-treatment MRI, integrated into an ML framework, can provide early prediction of 12-month tremor recurrence. This approach could help guide post-procedure decision-making, including consideration of early adjunctive interventions. Future studies should explore multiparametric models combining imaging with clinical and biohumoral markers, and SHAP-based interpretability may facilitate clinical adoption.

## Data Availability

The raw data supporting the conclusions of this article will be made available by the authors, without undue reservation.
