# Supplementary material for: Radiomic signatures from postprocedural MRI thalamotomy lesion can predict long-term clinical outcome in patients with tremor after MRgFUS: a pilot study
Source: Front Radiol. 2025 Nov 6;5:1683274. doi: 10.3389/fradi.2025.1683274 (PMC12631418; doi:10.3389/fradi.2025.1683274)
Supplement: Supplementary file 1 [file Supplementaryfile1.pdf]

Supplementary Materials for:

"Radiomic signatures from postprocedural MRI thalamotomy lesion can predict long-term clinical outcome in patients with tremor after MRgFUS: a pilot study"

Comparative Analysis

This supplementary section describes the evaluation pipeline used to compare four supervised learning models, a Support Vector Classifier (SVC) with a linear kernel, Logistic Regression (LR), Random Forest (RF), and Gradient Boosting classifier (GB), for the considered prediction task under a leave-one-out cross-validation (LOO CV) scheme. The performance metrics computed per iteration are aggregated across all folds to obtain the final estimates reported in Table S1.

Table S1: Comparative performance of the four classifiers under LOO CV. Best performances and model are in bold.

Model	Balanced Accuracy	MCC	Weighted F1
**Support Vector Classifier**	**0.720**	**0.356**	**0.737**
Logistic Regression	0.682	0.298	0.728
Random Forest	0.470	-0.081	0.694
Gradient Boosting classifier	0.502	0.006	0.718

Abbreviations: MCC = Matthews Correlation Coefficient.

Overall, the comparative analysis indicates that the linear SVC achieves the strongest and more per-class balanced performance.

Consistently, linear classifiers tend to perform well in this setting, as reflected by the competitive results obtained with LR. In contrast, tree-based models (RF and GB) are more prone to overfitting for this task, as reflected by comparatively lower discrimination metrics.
